# *Salmonella enterica* persister cells form unstable small colony variants after *in vitro* exposure to ciprofloxacin

**DOI:** 10.1038/s41598-019-43631-7

**Published:** 2019-05-10

**Authors:** Samara Paula Mattiello Drescher, Stephanie Wagner Gallo, Pedro Maria Abreu Ferreira, Carlos Alexandre Sanchez Ferreira, Sílvia Dias de Oliveira

**Affiliations:** 1 PUCRS, Escola de Ciências, Laboratório de Imunologia e Microbiologia, Porto Alegre, RS Brazil; 2 PUCRS, Escola de Ciências, Programa de Pós-graduação em Ecologia e Evolução da Biodiversidade, Porto Alegre, RS Brazil

**Keywords:** Antibacterial drug resistance, Biofilms, Clinical microbiology

## Abstract

Persistence phenotype and small colony variants (SCVs) can be part of a bacterial bet-hedging strategy for survival under environmental stresses, such as antimicrobial exposure. These phenotypes are of particular concern in persistent and relapsing infections, since cells resume to normal growth after cessation of the stressful condition. In this context, we found persisters and unstable SCVs as phenotypic variants of *Salmonella enterica* that were able to survive ciprofloxacin exposure. A high heterogeneity in persister levels was observed among *S*. *enterica* isolates grown under planktonic and biofilm conditions and exposed to ciprofloxacin or ceftazidime, which may indicate persistence as a non-multidrug-tolerant phenotype. Nevertheless, a comparable variability was not found in the formation of SCVs among the isolates. Indeed, similar proportions of SCV in relation to normal colony phenotype (NCP) were maintained even after three successive cycles of ciprofloxacin exposure testing colonies from both origins (SCV or NCP). Additionally, we found filamentous and dividing cells in the same scanning electron microscopy images from both SCV and NCP. These findings lead us to hypothesize that besides variability among isolates, a single isolate may generate distinct populations of persisters, where cells growing under distinct conditions may adopt different and perhaps complementary survival strategies.

## Introduction

*Salmonella enterica* comprises pathogens adapted to infect and survive inside human and animal epithelial and phagocytic cells^1,2^, including some non-host adapted serovars that are among the most important zoonotic pathogens worldwide. *Salmonella enterica* infection can result in diseases that range from gastroenteritis to enteric fevers. In the midst of this scenario, millions of foodborne outbreaks caused by *S*. *enterica* are reported every year, wherein the majority are due to consumption of food derived from animals^3^. Enteric fevers are life-threatening febrile illnesses requiring antibiotic therapy^4^, and fluoroquinolones, especially ciprofloxacin, are the chosen drugs. However, fluoroquinolones block DNA replication by inhibiting DNA gyrase and topoisomerase IV^5^ and are not suitable to treat infections in children and pregnant women^4^. Thus, in those cases, the treatment is performed using third-generation cephalosporins, such as ceftazidime, whose mechanism of action is the inhibition of peptidoglycan synthesis^6^.

Most *S*. *enterica* serovars are able to adhere to abiotic surfaces and persist in the environment for long periods, especially when growing as biofilms^7^. In fact, biofilms are recognized as major contributors to food processing cross-contamination due to the difficulty in removing them from contaminated surfaces. This makes them an important public health concern^8^. *In vivo*, biofilms can also prevent antimicrobial diffusion and block the entry of immune system components^9^. In addition, the higher bacterial survival levels in biofilms could be explained by the presence of persister cells^10^, a non-heritable phenotype that comprises a small subpopulation of cells derived from an isogenic bacterial culture, which displays high antibiotic tolerance by entering in a transient slow or non-growth state^10–12^. It is postulated that all bacteria can form persisters^13^, including *S*. *enterica*^14–17^, as well as archaea^18^ and fungi^19^. Persister cells can be stochastically formed in a microbial population, or induced by stressors such as antimicrobials. Indeed, persisters can survive exposure even to high levels of bactericidal antibiotics without undergoing any genetic change, unlike drug resistant cells^20^. Thus, eradication of persisters has become a challenge to avoid recurrent treatment failures and recalcitrance of chronic infections^10^. Molecular mechanisms behind persister cell formation have been studied, but they have not yet been fully elucidated. The trigger for persisters phenotype formation may involve a down or up-regulation of molecules related to stringent response^21^, energy production^22,23^, phosphate metabolism^24^, SOS response^25^ and toxin-antitoxin (TA) systems^17^, acting whether alone or overlapped^15,20^. *Salmonella* may form persisters in host macrophages when induced by vacuolar acidification and nutritional deprivation, and TA systems are presumed to be responsible for this microorganism’s physiological state^15^.

Another phenotypic switching found in response to harsh environments are the small colony variants (SCV)^26^. SCVs are characterized as slow-growing cells forming pin-prick-sized colonies^27^ that can revert to wild-type-like colonies^28,29^, or even be stably kept^26^, which enable survival to diverse environmental pressures, such as antimicrobial exposure^29^ and intracellular host defense^26^. Therefore, isogenic bacterial populations may present heterogeneous phenotypes such as persisters and SCVs.

We found persisters and unstable SCVs as phenotypic variants of *S*. *enterica* that were able to survive ciprofloxacin exposure. In addition, a high heterogeneity in the levels of persisters was observed among *S*. *enterica* isolates cultured under planktonic and biofilm conditions after ciprofloxacin or ceftazidime exposure, therefore not indicating persistence as a multidrug-tolerant phenotype. However, a similar variability was not found in the proportion of SCVs formed among the isolates, which was maintained even after successive treatments. Importantly scanning electron microscopy analysis allowed us to observe division septum and filamentous cells from both SCV and normal colony phenotype (NCP) images. Thus, our findings contribute to the characterization of these adaptive strategies to survive stressful environments, and may help to explain treatment failure and relapsing infections.

## Experimental Procedures

### Bacterial isolates

*Salmonella enterica* isolated between 1995 and 2012 from poultry by-product meals, poultry carcass, food, porcine faeces and food handler in Southern Brazil were used in this study as follows: *Salmonella* Schwarzengrund (n = 1), *Salmonella* Agona (n = 2), *Salmonella*. Infantis (n = 2) and *Salmonella* Enteritidis (n = 5) (Table 1). All isolates were stored at −80 °C in Trypticase Soy Broth (TSB) (BioBras, São Paulo, Brazil) with 20% glycerol.

**Table 1:** *Salmonella enterica* isolates and minimum concentration of ciprofloxacin (CIP) and ceftazidime (CAZ) required to inhibit their growth.

Isolates (ID)	Origin	MIC (µg/ml)	
		CIP	CAZ
*S*. Agona (S48)	Feathers meal	0.01	2
*S*. Agona (S79)	Meat meal	0.005	1
*S*. Enteritidis (152)	Ready-to-eat-food	0.005	0.5
*S*. Enteritidis (192)	Poultry carcass	0.01	1
*S*. Enteritidis (393)	Food handler	0.01	1
*S*. Enteritidis (4SA)	Porcine faeces	0.005	1
*S*. Enteritidis (S45)	Meat meal	0.005	2
*S*. Infantis (S02)	Meat meal	0.01	2
*S*. Infantis (S67)	Viscera meal	0.03	1
*S*. Schwarzengrund (S58)	Flesh and bones meal	0.005	1

MIC, minimum inhibitory concentration.

MIC breakpoints for CIP: ≤0.06 µg/ml, susceptible; 0.12–0.5 µg/ml, intermediate; ≥1 µg/ml, resistant. MIC breakpoints for CAZ: ≤4 µg/ml, susceptible; 8 µg/ml, intermediate; ≥16 µg/ml, resistant (CLSI Document M100-S28)^32^.

### Antimicrobial susceptibility

The ciprofloxacin (CIP) and ceftazidime (CAZ) (Sigma-Aldrich, St Louis, USA) minimum inhibitory concentrations (MIC) were determined by broth microdilution method, in triplicate^30^. The cut-off values were interpreted according to the Clinical and Laboratory Standards Institute guidelines^31^.

### Biofilm assay

All *S*. *enterica* isolates were evaluated with regard to biofilm formation in 96-well polystyrene plates. 1-μl aliquots of overnight cultures of each strain were adjusted to approximately 10^6^ colony-forming units per millilitre (CFU/ml). These were added in triplicate to wells containing 200 μl of fresh Luria Bertani (LB) broth [10 g/l tryptone (Kasvi, Roseto degli Abruzzi, Italy), 5 g/l yeast extract (Himedia, Mumbai, India) and 5 g/l NaCl (Nuclear, Diadema, Brazil), pH 7.2], and incubated for 48 h at 37 °C. Afterwards, wells were washed twice with phosphate-buffered saline (PBS) [8 g/l NaCl (Nuclear), 0.2 g/l KCl (Nuclear), 1.44 g/l Na_2_HPO_4_ (Nuclear) and 0.24 g/l KH_2_PO_4_ (Nuclear)] to remove planktonic cells, dried at 60 °C for 15 min, and then the biofilms were stained with 0.1% crystal violet for 5 min. After washing twice with PBS, wells were dried at 60 °C for 1 h and incubated with absolute ethanol for 15 min at room temperature. Wells containing only 200 µl of LB broth were used as negative control. Adherent cells were measured using a SpectraMax® 190 microplate reader (Molecular Devices, Sunnyvale, USA) at 570 nm. *Salmonella enterica* isolates were classified according to Stepanovic *et al*.^32^ as non-biofilm producers (OD ≤ OD_c_), weak biofilm producers (OD_c_ < OD ≤ 2OD_c_), moderate biofilm producers (2OD_c_ < OD ≤ 4OD_c_), and strong biofilm producers (4OD_c_ < OD). OD_c_ is the cut-off OD which was the mean OD plus three times the negative control standard deviations. *Salmonella* Typhimurium ATCC 14028 was used as a positive control for biofilm formation.

### Persister cell levels

**Figure 1:**
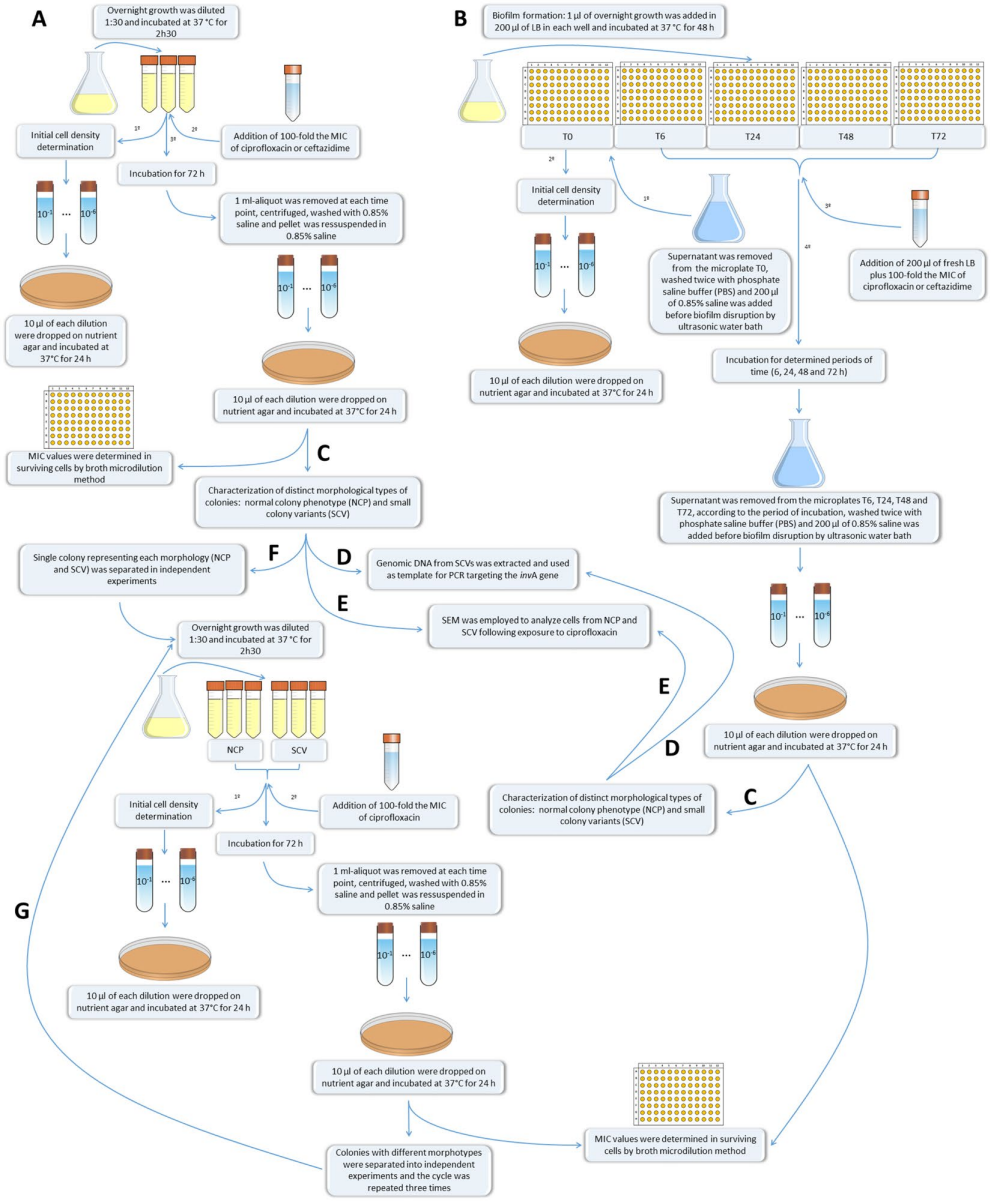
Flow diagram of experimental design for the *in vitro* evaluation of persistence in *Salmonella enterica*. (**A**) Evaluation of persister levels in planktonic culture at mid-exponential growth phase following exposure to ciprofloxacin or ceftazidime for 72 h. For the measurement of the surviving fractions, before addition of the antimicrobial, the initial cell density was determined by dilution and count of colonies on agar plate. After removal of the aliquot to determine initial cell density, cultures were exposed to 100-fold MIC of ciprofloxacin or ceftazidime for 72 h at room temperature, and 1 ml-aliquots were taken at 6, 12, 24, 48, and 72 h following the antimicrobial exposure for the count of colonies formed by the surviving cells. The minimum inhibitory concentration (MIC) of each antimicrobial was evaluated in the surviving cells by microdilution broth according to CLSI (2012). (**B**) Persister levels were also determined in biofilm after exposure to ciprofloxacin or ceftazidime. Firstly, isolates were grown as 48 h-biofilm on independent microplates according to the time point to be evaluated after the antimicrobial was added. Prior to the initial cell density determination, cells not adherent to the microplate T0 were discarded and biofilms were washed to be disrupted by ultrasonic water bath. After removal of the aliquot for the initial density determination, antimicrobial was added and cultures were incubated for 72 h at room temperature. At each time point (T6, T24, T48 and T72), biofilms were washed to be disrupted by ultrasonic water bath, and the following steps were performed according to described for the planktonic cultures. (**C**) Morphology of the colonies formed by persisters from both planktonic and biofilm cultures were analysed, and after exposure to ciprofloxacin were found small colony variants (SCV) in addition to the normal colony phenotype (NCP). (**D**) SCVs from both planktonic and biofilm growth were confirmed as *Salmonella* spp. by PCR targeting the *inv*A gene. (**E**) Morphology of the cells forming NCP and SCV from both planktonic and biofilm growth was analysed by scanning electron microscopy (SEM). (**F**) Stability of the SCV phenotype among persisters surviving after exposure to ciprofloxacin was evaluated, separately, in overnight cultures derived from both NCP and SCV. For this, the same procedures described for the evaluation of persisters in planktonic growth were employed and repeated for three consecutive cycles (**G**). The same procedures used to analyse stability of SCV originated from single colonies (SCV or NCP) were also employed using a pool of colonies derived from SCV or NCP. All assays were performed in three biological replicates, and data of CFU counts represent the mean of three replicates. Visual representations were taken from a free online source (clker.com) with the exception of microplates that were designed by the co-author S.P.M.D.

Persister cell levels were determined in planktonic and biofilm cultures after exposure to ciprofloxacin or ceftazidime according to the protocol described by Gallo *et al*.^33^, with some modifications (Fig. 1). To evaluate persister levels in planktonically growing cells, overnight cultures in LB broth were diluted 1:30 and incubated at 37 °C for 2 h 30 min until the mid-exponential growth phase (approximately 10^8^ CFU/ml) (Supplementary Fig. S1). Before antimicrobial exposure, the initial cell density was determined by diluting a 100 µl-aliquot until 10^−6^ in 0.85% saline and spotting 10 µl of each dilution in triplicate on nutrient agar (Oxoid, Hampshire, England), which was then incubated at 37 °C for 24 h. Afterwards, the mid-exponential growth phase cultures were exposed to antimicrobials at 100-fold MIC for each isolate at room temperature for 72 h (see Table 1). In order to determine the surviving fractions at 6, 12, 24, 48 and 72 h of antimicrobial exposure, 1 ml-aliquots were removed at each time, centrifuged at 7,200 rpm for 7 min, and the supernatants were discarded. The pellets were washed with 1 ml of 0.85% saline to remove antimicrobial residues. After washing, the pellets were resuspended in 1 ml of 0.85% saline that was diluted until 10^−6^, and 10 µl of each dilution were spotted on nutrient agar (Oxoid).

To determine the persister levels in biofilm, *S*. *enterica* isolates were grown in LB broth for 48 h at 37 °C using 96-well polystyrene plates. After this period, the culture medium containing non-adherent cells was removed and the biofilm was washed twice with PBS. The initial biofilm population density was evaluated by adding 200 µl of 0.85% saline to each well with subsequent disruption by an ultrasonic water bath (Ultrasonic Cleaner 1400 A, Unique, Indaiatuba, Brazil) for 10 min. For the determination of persistence levels, 200 µl of fresh LB broth containing 100-fold MIC of ciprofloxacin or ceftazidime were added to the 48 h-biofilms and incubated at room temperature until 72 h. At 6, 24, 48, and 72 h of exposure (evaluated in independent microplates), wells were washed twice with PBS, and 200 µl of 0.85% saline was added. Biofilms were disrupted by an ultrasonic water bath for 10 min. The supernatant containing dissociated adherent cells was removed and their quantification was performed as described for the planktonic cultures.

The survival cell fractions were calculated by dividing the number of remaining colonies counted by the number of colonies found before the antibiotic treatment. After a 72-h exposure to high concentrations of ciprofloxacin or ceftazidime, the MIC of each antimicrobial was determined again by broth microdilution^30^ in the surviving cells to exclude the selection of mutant resistant. All assays were performed in biological triplicate, and CFU count data were the means of three replicates.

### *Salmonella enterica* small colony variant (SCV)

**Figure 2:**
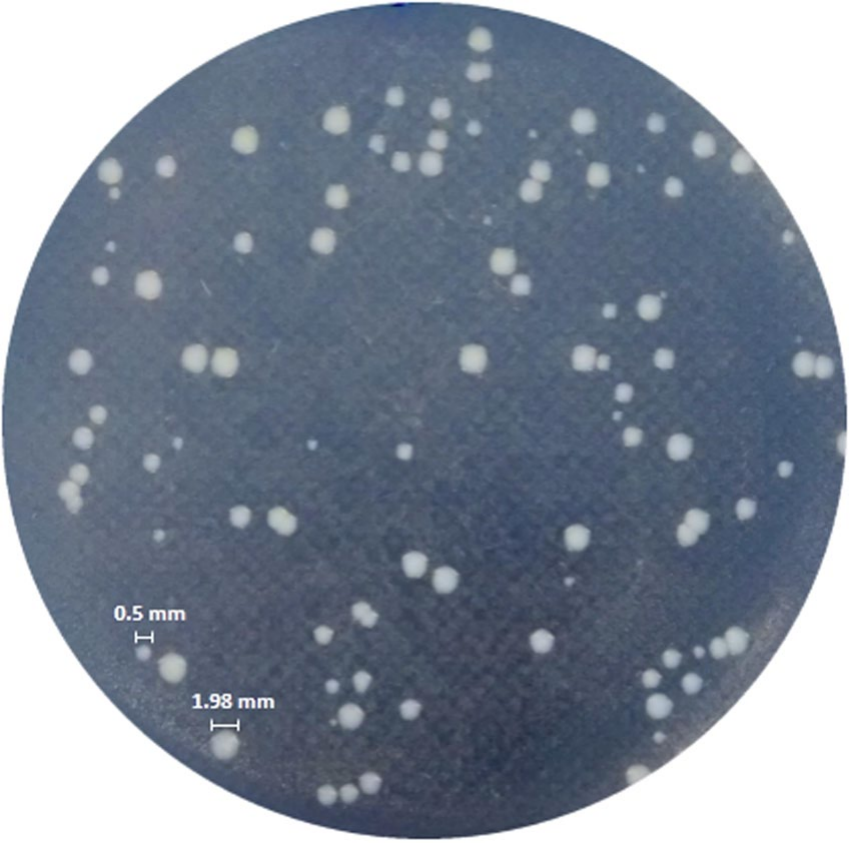
*Salmonella enterica* colony morphologies. After 72-h exposure to 100-fold MIC of ciprofloxacin, two different colony morphotypes were observed on nutrient agar during 24-h incubation at 37 °C, normal colony phenotype (NCP) and pinpoint colonies with reduced size, called small colony variants (SCV). The diameter of the colonies was measured using ImageJ software 1.8.0, represented here by an NCP of 1.98 mm and SCV of 0.5 mm.

Colonies formed by surviving cells after exposure to 100-fold MIC of ciprofloxacin or ceftazidime both in planktonic and biofilm cultures were morphologically analysed at all-time points (Fig. 1). *Salmonella enterica* SCVs were characterized by a maximum diameter of 0.5 mm, contrasting with around 2 mm diameter of the NCP on nutrient agar after 48-h incubation (Fig. 2). The cells from SCVs were also evaluated with regard to susceptibility to ciprofloxacin by broth microdilution^31^. Furthermore, to confirm SCVs as *S*. *enterica*, genomic DNA from each colony was extracted by boiling for 10 min^34^ and used as template for PCR targeting the *inv*A gene^35^.

The ability of SCVs to revert to a normal phenotype was evaluated by sub-culturing colonies from all isolates in a fresh nutrient agar without antimicrobials. Likewise, two isolates (*S*. Infantis S02 and *S*. Enteritidis 393) were used to investigate the stability of SCV phenotype after exposure to ciprofloxacin. For this, overnight cultures of each isolate were diluted 1:30 with fresh LB broth and cultured at 37 °C for 2 h 30 min until the mid-exponential growth phase. Afterwards, cultures were incubated with ciprofloxacin at 100-fold MIC for 72 h at room temperature, and spotted on nutrient agar. Surviving cells from one NCP and one SCV were separated into independent experiments. Each colony was grown separately overnight in a fresh LB broth, diluted 1:30 with fresh LB broth, cultured at 37 °C for 2 h 30 min, exposed again to ciprofloxacin at 100-fold MIC for 72 h at room temperature, and spotted on nutrient agar (cycle 1). Surviving cells from one NCP and one SCV were again separated into independent experiments and the assay was repeated two more times (cycles 2 and 3) as described for cycle 1. All tests were performed in three independent biological replicates. This same assay used to evaluate one colony of each morphology was performed using a pool of ten each of NCPs or SCVs. In each cycle, susceptibility to ciprofloxacin was re-evaluated by broth microdilution^30^.

### Scanning electron microscopy (SEM)

SEM was employed to analyse *S*. Enteritidis 393 cells from normal and small colonies cultured under planktonic and biofilm conditions exposed to ciprofloxacin. As mentioned above, after exposure to antimicrobials for 72 h, aliquots of cultures grown under each condition were removed, plated on nutrient agar, and grown at 37 °C for 24 h. Afterwards, NCPs and SCVs, 10 of each, were gently collected, inoculated in 1 ml of 0.85% saline, and centrifuged at 7,200 rpm for 7 min. The supernatants were removed and the pellets were immediately fixed by immersion in 2.5% glutaraldehyde and 0.1 M phosphate buffer (pH 7.2–7.4) for one week. Then, cells were adhered on 18-mm glass coverslips previously coated with poly-L-lysine. The material was washed thrice with phosphate buffer, dehydrated with acetone, and desiccated to remove the acetone, followed by gold metallization. The images were observed with a Field Emission Scanning Electron Microscope (Inspect F50, FEI Company Inspect, Eindhoven, Netherlands) at the Central Laboratory of Microscopy and Microanalysis (LabCEMM) of PUCRS.

### Statistical analysis

Surviving fractions from planktonic or biofilm cultures after treatment with antimicrobials for 72 h were compared using an analysis of variance (ANOVA) with permutations (9,999 bootstrap iterations in all tests), and repeated measures ANOVA when applicable. Analyses were carried out with pooled mean values of all isolates, and considering each isolate separately. The same analyses were performed to compare the SCV ratios found for both culture conditions and antimicrobial exposures and to evaluate the stability of the SCV phenotype during successive cycles of exposure to ciprofloxacin. Pairwise comparisons between persister fractions obtained from different serovars, as well as the SCV ratios found in different serovars, were employed using Tukey’s post-hoc test after ANOVA with permutations. All analyses were conducted in the statistical platform R^36^ using ‘ImPerm’ package^37^. We considered *p*-values ≤ 0.05 as significant.

## Results

### Biofilm intensity and minimum inhibitory concentration to ciprofloxacin and ceftazidime

All *S*. *enterica* isolates were characterized as weak biofilm producers after growth in polystyrene microplates for 48 h, and cell densities ranged from 6.1 × 10^6^ to 3.9 × 10^7^ CFU (Supplementary Tables S1 and S2). The MIC values ranged from 0.005 to 0.01 µg/ml and 0.5 to 2 µg/ml, for ciprofloxacin and ceftazidime, respectively (Table 1), which characterized all isolates as susceptible to both antimicrobials.

### Different persister levels were found in *S. enterica* isolates after ciprofloxacin or ceftazidime exposure

Persister cells were detected in all *S*. *enterica* isolates after 72-h exposure to high concentrations of ciprofloxacin or ceftazidime in both planktonic and biofilm cultures. In order to assure the presence of *S*. *enterica* persisters and not of antibiotic-resistant mutants, a new susceptibility test was performed after all persister assays with the remaining 72-h cells and no difference in MIC values was detected.

**Figure 3:**
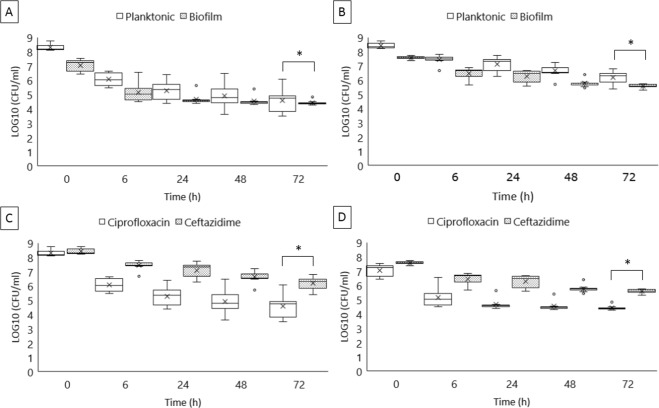
Persister fractions of *Salmonella enterica* after exposure to antimicrobials for 72 h. Box plots representing the average and variance of all isolates (*n* = 10) cultured under planktonic and biofilm conditions at each time evaluated after exposure to (**A**) 100-fold MIC of ciprofloxacin or (**B**) ceftazidime, as well as the persister fractions after exposure to ciprofloxacin or ceftazidime found in the isolates growing as (**C**) planktonic culture and (**D**) biofilm. Surviving fractions after 72 h-exposure to antimicrobials were compared by ANOVA with permutation, considering *p*-values ≤ 0.05 (*) as significant.

Treatments with 100-fold MIC of ciprofloxacin or ceftazidime for 72 h resulted in distinct persister fractions (*p* < 0.05) in planktonically grown cells ranging from 0.0020% to 0.2252% (Fig. 3A,C and Supplementary Table S1) and 0.1466% to 1.6755% (Fig. 3B,C and Supplementary Table S2), respectively. In the same context, persister fractions from biofilms after a 72-h treatment with ciprofloxacin or ceftazidime ranged from 0.0694% to 0.9378% (Fig. 3A,D and Supplementary Table S1) and 0.6076% to 1.5869% (Fig. 3B,D and Supplementary Table S2), respectively. All *S*. *enterica* isolates, except for three *S*. Enteritidis (192, 4SA, and S45) grown as biofilms, had significantly different persister levels (*p* < 0.05) when exposed to the distinct antimicrobials (Supplementary Table S3). Furthermore, a high heterogeneity in persister levels was found among *S*. *enterica* isolates when cultured under the same conditions and exposed to a same antimicrobial, especially planktonically grown cells exposed to ciprofloxacin (Fig. 3A,C and Supplementary Tables S1 and S2).

### Biofilms presented higher persister levels than planktonic cultures

**Figure 4:**
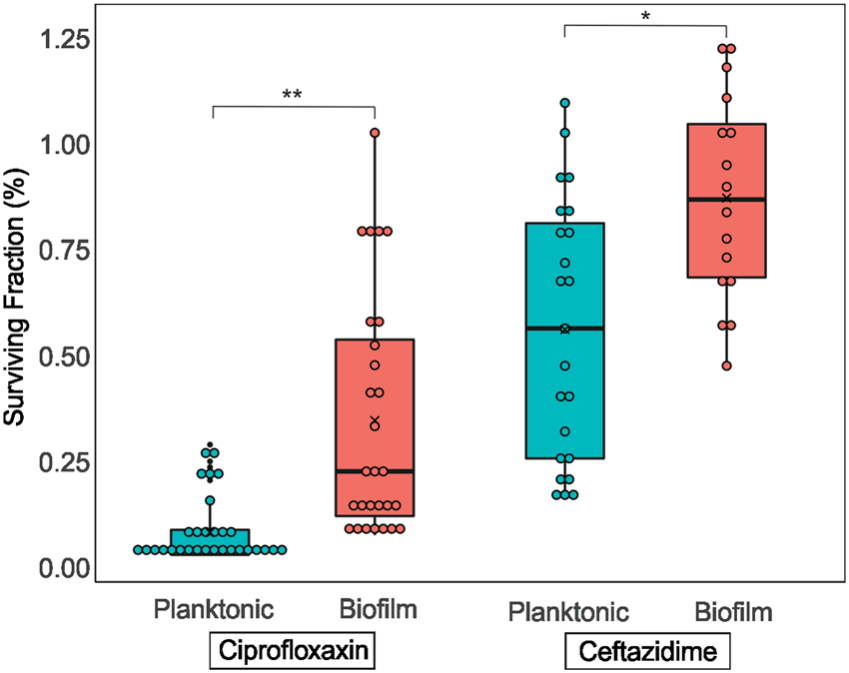
Comparison among persister fractions obtained from all *Salmonella enterica* isolates in planktonic and biofilm cultures exposed to ceftazidime or ciprofloxacin. In each box, bold horizontal lines and ‘x’ letters represent medians and mean values, respectively. Results from the analysis of variance with permutation are represented as *p*-values ≤ 0.05 (*) and ≤0.001 (**).

Taking together the persister fractions from all isolates, it was possible to notice higher levels of persisters in biofilms compared to planktonic cultures, and in both of those exposed to ciprofloxacin (*p* < 0.001) or ceftazidime (*p* < 0.05) (Fig. 4). Indeed, in some isolates, the persister levels found in biofilms after ciprofloxacin exposure were up to 140-fold higher than those detected in planktonic counterparts (Supplementary Table S1).

### Levels of persisters after ceftazidime exposure were not affected by serovar regardless of culture condition

Persister levels in isolates from different serovars cultured under both conditions after exposure to ceftazidime or ciprofloxacin were compared, and no difference (*p* > 0.05) was found among serovars after ceftazidime treatment, as well as in biofilms exposed to ciprofloxacin (Supplementary Table S4). On the other hand, persister levels from planktonic cultures exposed to ciprofloxacin varied depending on the serovar (*p* < 0.01), except when *S*. Enteritidis was compared with *S*. Infantis (*p* = 0.7364). In addition, we found significantly different persister levels among *S*. Enteritidis isolates when comparing both culture conditions regardless of the antimicrobial used; this was also observed among *S*. Infantis isolates (*p* < 0.05) (Supplementary Table S5).

### SCVs were found among *S*. *enterica* tolerant to ciprofloxacin

After ciprofloxacin exposure, colonies formed by surviving cells were morphologically analysed and SCVs could be seen from all *S*. *enterica* isolates (Fig. 2). All isolates showed similar ratios of SCVs in relation to the total number of colonies formed by persisters (*p* > 0.05) (Supplementary Fig. S2). However, when comparing colonies of persisters from all isolates in planktonic cultures with biofilms, SCVs were observed in higher proportion in planktonic cultures (Supplementary Fig. S3) (*p* < 0.05). On the other hand, similar ratios of SCVs were detected in both culture conditions for the same isolate (Supplementary Table S6). All SCVs reverted to a wild-type-like phenotype after sub-culturing in a medium without an antimicrobial. SCVs were confirmed as *Salmonella* spp. by the presence of the *inv*A gene (Supplementary Fig. S4), and susceptibility to ciprofloxacin was maintained, since no difference between MIC values from NCPs and SCVs were detected. In groups of isolates from the same serovar, we did not find significant differences between each group in the ratios of SCVs to total colony numbers formed by persisters (Supplementary Fig. S5). Culture conditions also did not have a significant influence on the ratio of SCVs formed in each serovar (Supplementary Table S7). SCVs could not be observed in ceftazidime assays even after 48-h incubation.

**Figure 5:**
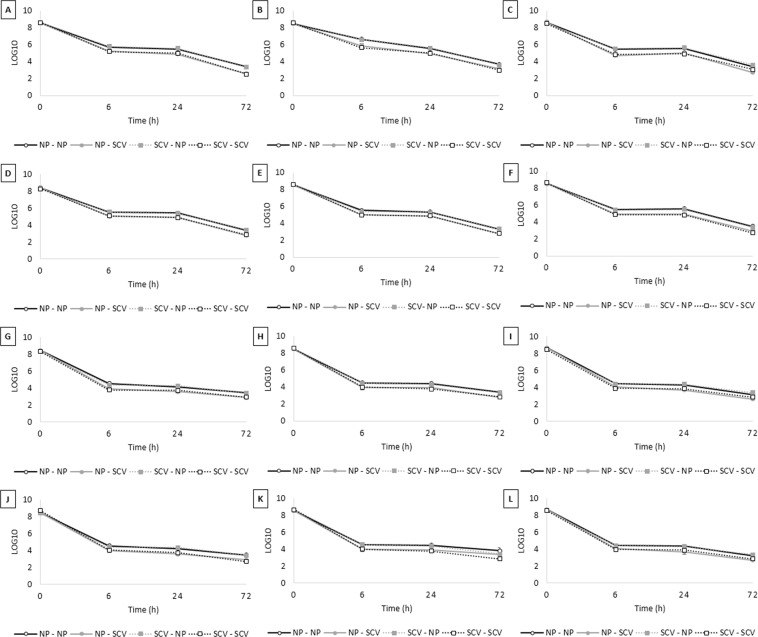
Small colony variants (SCV) phenotype evaluation throughout three consecutive cycles. Overnight culture was diluted 1:30, grew until mid-log phase and treated with ciprofloxacin 100-fold MIC for 72-h. The surviving cells forming SCV and normal colony phenotype (NCP) were separated in different experiments. The procedure was repeated three times and at the end of each cycle, SCV was obtained from NCP (NCP-SCV) or SCV (SCV-SCV) and NCP was also obtained from NCP (NCP-NCP) or SCV (SCV-NCP). (**A**–**F**) *Salmonella* Infantis: cycle one to three performed with (**A**–**C**) only one colony or (**D**–**F**) pool of colonies. (**G**–**L**) *Salmonella* Enteritidis: cycle one to three performed with (**G**–**I**) only one colony or (**J**–**L**) pool of colonies. The values are average of three biological with three technical replicates and bars indicate the standard error.

Throughout three cycles, regardless if the analysis was performed from a single colony or from a pool of ten colonies, or whether originating from SCVs or NCPs, there was no significant difference between persister fractions forming SCVs after 72-h exposure to ciprofloxacin (*p* > 0.05). The same findings were observed in isolates belonging to different serovars (*S*. Infantis and *S*. Enteritidis). Therefore, a stable SCV phenotype was not selected throughout three cycles. Interestingly, when analysing the persister levels during 72-h ciprofloxacin exposure in each cycle for both isolates, we detected similar fractions from cells growing as SCVs or NCPs, regardless of their source (Fig. 5 and Table 2).

**Table 2:** Small colony variants (SCV) persister fractions obtained from cells growing as SCVs or as normal colony phenotype (NCP) during three ciprofloxacin exposure cycles derived from a single colony or a pool of ten colonies.

Isolate	Single colony or pool of colonies	Source (NCP or SCV)	Cycle	SCV persister fraction (%)
*S*. Infantis (S02)	Single colony	SCV	1	0.00008
	Single colony	SCV	2	0.00026
	Single colony	SCV	3	0.00040
	Single colony	NCP	1	0.00010
	Single colony	NCP	2	0.00045
	Single colony	NCP	3	0.00011
	Pool	SCV	1	0.00037
	Pool	SCV	2	0.00015
	Pool	SCV	3	0.00011
	Pool	NCP	1	0.00090
	Pool	NCP	2	0.00018
	Pool	NCP	3	0.00030
*S*. Enteritidis (393)	Single colony	SCV	1	0.00043
	Single colony	SCV	2	0.00019
	Single colony	SCV	3	0.00024
	Single colony	NCP	1	0.00030
	Single colony	NCP	2	0.00022
	Single colony	NCP	3	0.00009
	Pool	SCV	1	0.00036
	Pool	SCV	2	0.00017
	Pool	SCV	3	0.00021
	Pool	NCP	1	0.00030
	Pool	NCP	2	0.00074
	Pool	NCP	3	0.00010

### Cells from SCV and NCP showed similar size, division septum and filamentation

**Figure 6:**
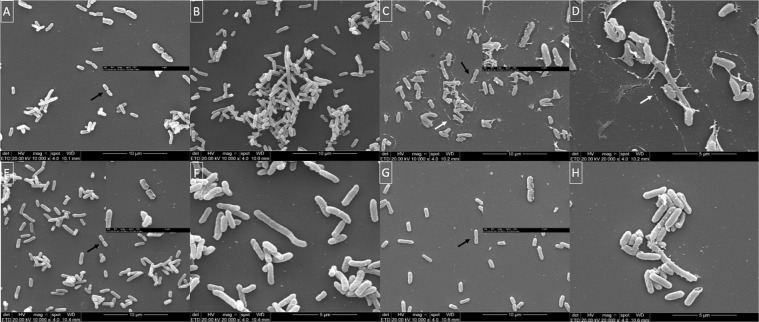
Scanning electron microscopy of *Salmonella* Enteritidis (393) forming small colony variants (SCV) and wild-type-like phenotype colonies after exposure to 100-fold MIC of ciprofloxacin for 72 h. In cells from both (**A**,**B**,**E**,**F**) wild-type-like phenotype and (**C**,**D**,**G**,**H**) SCV derived from (**A**–**D**) planktonic and (**E**–**H**) biofilm cultures were observed septum division (insets), (**B**,**D**,**F**,**H**) filamentation, and similar size between wild-type-like phenotype and SCV (**A**) (1.129–1.155 µm), (**C**) (1.285–1327 µm), (**E**) (1.057–1.041 µm) and (**G**) (1.275–1.158 µm). (**C**,**D**) White arrow indicates the extracellular substance in SCVs obtained from planktonic culture and black arrows indicate septum division.

SEM was employed to evaluate morphology of cells from SCV and NCP grown in planktonic and biofilm conditions (Fig. 6). Regardless of the culture condition, a similar size was observed in cells from both SCVs and NCPs (Fig. 6A,C,E,G). Interestingly, in both SCVs and NCPs cultured in planktonic and biofilm condition, we found filamentous cells (Fig. 6B,D,F,H) concurrent with cells showing septum division (Fig. 6A,C,E,G). Furthermore, an extracellular substance was noticed circumventing SCVs cells obtained from planktonic culture (Fig. 6C,D), and filamentous cells were observed in SCVs and NCPs from both planktonic and biofilm cultures.

## Discussion

Bacteria can continuously face unpredictable stresses, such as host immune defence; starvation; temperature, oxygen, and pH alterations, and antimicrobial action^38^. The phenotypic switching that occurs in a small number of individuals within isogenic populations can be an essential adaptability strategy that is adopted by many microorganisms to survive different challenges^28,38^. Persisters and SCVs comprise phenotypic variants able to survive a hostile environment and can resume normal growth after the stressful condition has ceased^10,28^.

In this paper, we showed that isolates from four distinct *S*. *enterica* serovars were able to generate persisters after exposure to both antimicrobials tested. However, we did not find correlations between persister levels and *S*. *enterica* serovars, especially when ceftazidime was employed; however, a fluctuation in persister fractions among serovars was noticed in planktonic cultures exposed to ciprofloxacin. Nevertheless, different persister levels were found after exposure of isolates to antimicrobials with distinct action mechanisms. These findings led us to hypothesize that a single isolate generates distinct populations of persisters, each one with particular mechanisms to tolerate the lethal effects of different bactericidal antibiotics. Thus, the classical paradigm of a multidrug-tolerance phenotype because of antimicrobial ineffectiveness^12,39^ may not be present in all persister cells.

*S*. *enterica* persister levels also varied with regard to the culture conditions. Indeed, higher levels of surviving cells were detected in biofilms when compared to their planktonic counterparts, especially after ciprofloxacin exposure. It is important to highlight that the planktonic cells evaluated here were from a mid-exponential phase, since levels of persisters from bacterial cells growing in a stationary phase have been described as similar to or even higher than those found in biofilms^40^. Bacteria growing in biofilms can face stressful conditions related to persistence, such as starvation^41^, oxygen deprivation^42^ and limited metabolic flux^43^, triggering a stringent response, which, in turn, may activates the SOS response^44^. The SOS response allows survival after exposure to β-lactams and fluoroquinolone antibiotics^25,45^, and has been proposed as necessary for biofilm ofloxacin tolerance^46^.

Quorum sensing had also been associated with persister formation in biofilms^47^; however, we found that both biofilm and planktonic cultures with higher initial densities did not have the highest persister levels (Supplementary Tables S1 and S2), which we had previously reported in *Acinetobacter calcoaceticus*-*baumannii*^33^. Additionally, initial cell density, i.e., the population before antibiotic exposure, was higher in planktonic cultures than in biofilms. Therefore, we were not able to corroborate that quorum sensing is playing a major role in the generation of persisters in biofilms; once we detected up to a 10-fold variation in biofilm persister levels when initial cell densities were similar. Indeed, an important aspect to take into account is the physiological states of cells growing in different conditions, which would be involved with the ability to respond to stresses and transport substances across membranes^48^.

In addition to isolates with distinct behaviours that are related to persister levels when exposed to different antimicrobials and/or cultured under different conditions, heterogeneity was observed among isolates facing a same situation, which highlights a wide individual variation in antimicrobial tolerance, as also described in other bacteria^33,41,49,50^.

Phenotypic switching to SCVs has also been recognized as a strategy for antimicrobial tolerance^51^. Here, we reported SCVs among persisters surviving after ciprofloxacin treatment in all *S*. *enterica* isolates, and a stable SCV phenotype was not found even in three consecutive cycles of ciprofloxacin exposure, regardless of whether the origin of the colony was small or normal, since all SCVs reverted to the wild-type-like phenotype when sub-cultured under stress-free conditions. This can indicate that SCVs, like persisters, represent a transient phenotype originating from stress responses and possibly coordinated by epigenetic changes^52,53^. Furthermore, we also confirmed that the formation of persisters in *S. enterica* is a non-heritable mechanism, since the fractions of persisters remained approximately the same during repeated cycles.

All unstable SCVs we detected maintained the same ciprofloxacin MIC values of their ancestors, as already described in other studies^24,27^. However, SCVs have been reported to be less susceptible to aminoglycosides and β-lactams antimicrobials^54–56^, especially in stable SCVs, which may be due to mutations in genes involved in pathways required for the antimicrobial actions independent of those involved in the small colony size phenotype^57^.

Despite the wide variation of persister levels found between the isolates, the ratios of SCV:total colonies were not different among them. Another important aspect to be highlighted was the detection of more SCVs in planktonic cultures than in biofilms, unlike what is usually found in persisters, which allow us to speculate that cells growing under distinct conditions may adopt different and perhaps complementary survival strategies. Nevertheless, no fluctuation in SCV rates among serovars could be seen, leading us to assume that serovars do not influence in SCV rates.

We also investigated the cell morphology of cells forming SCVs and NCPs, since SCVs from *Staphylococcus* spp. have been described as cells with different sizes, smaller or larger, when compared to those of NCPs^52,58^. However, we observed similar shapes and sizes when comparing all cells, regardless of the culture conditions (Fig. 6A,C,E,G). Nevertheless, filamentous cells were seen among cells forming both SCVs and NCPs. These have been reported in *E*. *coli* after exposure to ciprofloxacin due to the inhibition of cell division resulting from the induction of SOS response and raise of DNA-repair capability^59,60^. In opposite, several cells showed septum division, which lead us to suggest that different behaviours can be found among cells forming distinct colonies morphologies, where cells may exhibit metabolic activity at different levels resulting in different growing speeds.

Both SCV and persisters are thought to be part of a bacterial bet-hedging strategy for the survival under stress. So, could SCVs comprise a phenotypic variant of persisters characterized by slow growth? If we consider these phenotypes as independent variants in *S*. *enterica* that randomly generate unstable SCV regardless of antibiotic exposure, we should also have found SCV after exposure to ceftazidime, which did not happen. Can we postulate that the diversity of strategies may be greater or that there is an overlap of physiological strategies depending on the challenging stress? Thus, elucidating the mechanisms involved in phenotypic switching of *S*. *enterica* isolates in an isogenic population is essential for the development of methods for an effective treatment of chronic infections, which may be of special concern in infections caused by invasive *Salmonella* serovars.

## Supplementary information

Supplementary information
