# Supplementary material for: Salmonella enterica persister cells form unstable small colony variants after in vitro exposure to ciprofloxacin
Source: Sci Rep. 2019 May 10;9:7232. doi: 10.1038/s41598-019-43631-7 (PMC6510897; doi:10.1038/s41598-019-43631-7)
Supplement: Supplementary file 1 — Supplementary information [file 41598_2019_43631_MOESM1_ESM.pdf]

Salmonella enterica persister cells form unstable small colony variants after *in vitro* exposure to ciprofloxacin

Samara Paula Mattiello Drescher, Stephanie Wagner Gallo, Pedro Maria Abreu Ferreira, Carlos Alexandre Sanchez Ferreira, Sílvia Dias de Oliveira*

Supplementary Table 1. Colony forming units (CFU) from *Salmonella enterica* isolates after exposure to ciprofloxacin for 72 h in planktonic and biofilm cultures.

Isolate	Planktonic						Biofilm					
	**0h***	**6h**	**24h**	**48h**	**72h**	**Persister fractions (%)**	**0h**	**6h**	**24h**	**48h**	**72h**	**Persister fractions (%)**
S. Agona (S48)	1.28E+08[†]± 1.73E+06[‡]	3.78E+06± 1.17E+06	3.11E+05± 8.40E+04	1.36E+05± 1.03E+04	2.22E+05± 5.08E+04	0.1737	1.44E+07± 1.96E+06	6.66E+04± 5.77E+03	4.22E+04± 5.08E+03	2.55E+04± 3.87E+03	1.89E+04± 6.97E+03	0.1353
S. Agona (S79)	1.32E+08± 8.54E+06	6.11E+05± 1.68E+05	2.58E+05± 2.15E+04	3.11E+05± 6.97E+04	7.22E+04± 1.26E+04	0.0552	2.67E+06± 3.35E+05	3.11E+04± 6.97E+03	2.33E+04± 3.35E+03	2.45E+04± 6.93E+03	1.78E+04± 5.08E+03	0.6849
S. Enteritidis (152)	1.47E+08± 2.08E+07	3.33E+05± 1.21E+05	2.78E+04± 5.08E+03	5.67E+03± 1.34E+03	4.67E+03± 3.35E+02	0.0032	3.33E+07± 1.34E+07	3.33E+05± 5.77E+04	4.56E+04± 1.50E+04	3.44E+04± 5.10E+03	2.67E+04± 3.35E+03	0.0892
S. Enteritidis (192)	1.61E+08± 1.15E+07	4.34E+05± 1.53E+05	2.44E+04± 5.10E+03	4.11E+03± 7.68E+02	3.22E+03± 3.87E+02	0.0020	2.11E+07± 1.35E+07	3.78E+06± 1.65E+06	4.22E+05± 1.26E+05	2.33E+05± 6.65E+04	6.89E+04± 7.68E+03	0.4007
S. Enteritidis (393)	1.54E+08± 2.31E+06	3.00E+05± 6.70E+04	4.00E+04± 6.70E+03	2.22E+04± 5.08E+03	1.67E+04± 3.35E+03	0.0108	2.33E+07± 1.00E+07	1.56E+05± 1.96E+04	4.33E+04± 1.34E+04	2.89E+04± 8.40E+03	2.45E+04± 3.87E+03	0.1251
S. Enteritidis (4SA)	2.89E+08± 1.02E+08	2.33E+06± 3.35E+05	2.00E+05± 8.83E+04	4.11E+04± 1.26E+04	4.44E+04± 1.96E+03	0.0165	2.67E+06± 3.35E+05	4.56E+04± 1.26E+04	3.56E+04± 5.10E+03	2.33E+04± 6.65E+03	2.44E+04± 5.10E+03	0.9378
S. Enteritidis (S45)	4.89E+08± 1.65E+08	4.22E+06± 7.74E+05	1.53E+06± 2.31E+05	8.00E+05± 3.30E+04	8.33E+04± 3.35E+03	0.0188	3.66E+06± 1.15E+06	3.78E+05± 7.74E+04	3.11E+04± 5.10E+03	2.89E+04± 9.64E+03	2.55E+04± 1.07E+04	0.7879
S. Infantis (S02)	1.30E+08± 8.89E+06	4.78E+05± 1.02E+05	5.78E+04± 6.93E+03	5.00E+04± 1.15E+04	3.22E+03± 6.93E+02	0.0025	3.22E+07± 6.93E+06	1.55E+05± 3.87E+04	4.11E+04± 5.10E+03	3.11E+04± 1.34E+04	2.22E+04± 8.40E+03	0.0694
S. Infantis (S67)	1.27E+08± 5.77E+06	1.78E+06± 8.40E+05	5.78E+05± 5.08E+04	7.44E+04± 5.10E+03	6.67E+04± 1.20E+04	0.0530	2.33E+07± 6.65E+06	4.11E+04± 1.26E+04	3.44E+04± 5.10E+03	2.00E+04± 3.30E+03	2.22E+04± 8.40E+03	0.0989
S. Schwarzengrund (S58)	5.45E+08± 6.93E+07	4.44E+06± 1.96E+05	2.48E+06± 1.50E+05	2.92E+06± 1.01E+05	1.21E+06± 7.21E+04	0.2252	6.45E+06± 1.57E+06	3.11E+04± 6.97E+03	2.33E+04± 3.35E+03	2.45E+04± 6.93E+03	1.78E+04± 5.08E+03	0.5195

*CFU counts from culture before adding antimicrobial. The persister fractions at each time point should take into account the value of CFU in 0h for each culture condition.

† Data of CFU counts represent the average of three biological and three technical replicates.

‡ Standard Deviation

Supplementary Table 2. Colony forming units (CFU) from *Salmonella enterica* isolates after exposure to ceftazidime for 72 h in planktonic and biofilm cultures.

Isolate	Planktonic						Biofilm					
	0h*	6h	24h	48h	72h	Persister fractions (%)	0h	6h	24h	48h	72h	Persister fractions (%)
S. Agona (S48)	1.78E+08†± 5.08E+07‡	2.67E+07± 8.79E+06	4.33E+06± 8.79E+05	3.00E+06± 3.30E+05	5.55E+05± 6.93E+04	0.3279	3.67E+07± 1.34E+07	4.22E+06± 5.08E+05	1.44E+06± 5.10E+05	3.56E+05± 1.26E+05	3.56E+05± 1.26E+05	1.0613
S. Agona (S79)	1.78E+08± 1.91E+07	5.00E+07± 1.20E+07	2.45E+07± 6.93E+06	1.66E+07± 5.77E+06	3.00E+06± 6.70E+05	1.6755	2.33E+07± 3.35E+06	4.55E+05± 1.07E+05	3.78E+05± 1.02E+05	4.33E+05± 1.00E+05	3.00E+05± 3.30E+04	1.3097
S. Enteritidis (152)	4.56E+08± 1.39E+08	6.11E+07± 6.97E+06	5.44E+07± 1.26E+07	1.67E+07± 3.35E+06	6.11E+06± 1.02E+06	1.4639	4.44E+07± 8.36E+06	5.00E+06± 1.76E+06	2.44E+06± 8.36E+05	6.00E+05± 8.83E+04	5.11E+05± 8.40E+04	1.2007
S. Enteritidis (192)	1.67E+08± 3.35E+07	4.55E+06± 6.93E+05	1.89E+06± 5.10E+05	5.11E+05± 5.10E+04	2.44E+05± 5.10E+04	0.1466	5.44E+07± 1.02E+07	6.44E+06± 1.17E+06	3.67E+06± 1.21E+06	2.44E+06± 8.36E+05	5.45E+05± 1.35E+05	1.0574
S. Enteritidis (393)	5.56E+08± 1.17E+08	2.78E+07± 8.40E+06	2.67E+07± 8.79E+06	3.11E+06± 5.10E+05	2.33E+06± 6.65E+05	0.4275	5.45E+07± 1.35E+07	7.11E+06± 1.26E+06	4.33E+06± 6.65E+05	5.67E+05± 6.65E+04	3.22E+05± 6.93E+04	0.6076
S. Enteritidis (4SA)	1.78E+08± 3.87E+07	2.44E+07± 5.10E+06	5.44E+06± 1.54E+06	3.67E+06± 6.65E+05	1.44E+06± 5.10E+05	0.8056	3.56E+07± 8.36E+06	1.33E+06± 3.35E+05	5.44E+05± 1.02E+05	4.89E+05± 1.07E+05	4.22E+05± 1.02E+05	1.2078
S. Enteritidis (S45)	3.89E+08± 3.81E+07	3.89E+07± 5.10E+06	1.89E+07± 6.97E+06	3.67E+06± 1.00E+06	2.89E+06± 5.10E+05	0.7450	4.11E+07± 8.40E+06	5.11E+06± 6.97E+05	4.45E+06± 1.07E+06	7.11E+05± 4.99E+04	5.00E+05± 8.83E+04	1.2283
S. Infantis (S02)	1.89E+08± 1.91E+07	2.22E+07± 9.58E+06	4.25E+06± 1.55E+06	3.66E+06± 5.77E+05	3.78E+05± 8.40E+04	0.1989	3.11E+07± 7.68E+06	5.00E+06± 1.20E+06	4.78E+06± 1.02E+06	5.78E+05± 6.93E+04	3.45E+05± 3.87E+04	1.2471
S. Infantis (S67)	2.22E+08± 6.93E+07	4.00E+07± 8.83E+06	3.11E+07± 8.40E+06	7.11E+06± 3.81E+05	1.78E+06± 5.08E+05	0.8027	2.45E+07± 6.93E+06	5.11E+05± 1.68E+05	4.33E+05± 3.35E+04	2.78E+05± 5.08E+04	2.00E+05± 3.32E+04	0.8756
S. Schwarzengrund (S58)	3.45E+08± 1.07E+08	3.00E+07± 8.83E+06	2.67E+07± 6.65E+06	7.22E+06± 8.40E+05	4.78E+06± 6.93E+05	1.4668	3.56E+07± 5.10E+06	4.67E+06± 8.75E+05	3.44E+06± 8.36E+05	5.89E+05± 8.40E+04	5.67E+05± 1.21E+05	1.5869

*CFU counts from culture before adding antimicrobial. The persister fractions at each time point should take into account the value of CFU in 0h for each culture condition.

† Data of CFU counts represent the average of three biological and three technical replicates.

‡ Standard Deviation

Supplementary Table 3. Analysis of variance with permutation (PERM-ANOVA) of persister fractions obtained from each *Salmonella enterica* isolate in planktonic and biofilm cultures exposed to ceftazidime or ciprofloxacin.

**Ciprofloxacin vs Ceftazidime**						
**Isolate**	**Biofilm**			**Planktonic**		
	**Sum of squares**	**F-statistic**	**p-value**	**Sum of squares**	**F-statistic**	**p-value**
152	0.00018	21.52000	0.00974	0.00032	16.84000	0.01481
192	0.00006	5.35700	0.08163	0.00001	1639.00000	0.00001
393	0.00003	29.01000	0.00574	0.00002	34.23000	0.00425
4SA	0.00001	1.34200	0.31120	0.00009	61.76000	0.00141
S02	0.00020	20.73000	0.01039	0.00001	128.10000	0.00034
S45	0.00002	2.18100	0.21380	0.00008	76.54000	0.00094
S48	0.00012	12.04000	0.02560	0.00001	7.98600	0.04754
S58	0.00017	36.40000	0.00380	0.00023	20.20000	0.01087
S67	0.00009	17.34000	0.01410	0.00008	1642.00000	0.00001
S79	0.00005	7.88600	0.04841	0.00039	132.40000	0.00036

Supplementary Table 4. Comparison between persister fractions from each culture conditions and antimicrobial exposure in each group of *Salmonella enterica* serovars employing Tukey's test.

		Agona	Enteritidis	Infantis	Schwarzengrund
**Biofilm Ceftazidime**	Agona		0.88820	0.93190	0.40860
	Enteritidis	1.01800		1.00000	0.12090
	Infantis	0.84510	0.00824		0.19110
	Schwarzengrund	2.23000	3.27100	2.92000	
		Agona	Enteritidis	Infantis	Schwarzengrund
**Planktonic Ceftazidime**	Agona		0.70360	0.39820	0.62630
	Enteritidis	1.52900		0.84150	0.15580
	Infantis	2.25700	1.16800		0.08126
	Schwarzengrund	1.71100	3.08100	3.55400	
		Agona	Enteritidis	Infantis	Schwarzengrund
**Biofilm Ciprofloxacin**	Agona		0.98610	0.39540	0.97120
	Enteritidis	0.48170		0.13530	0.99560
	Infantis	2.26500	3.18800		0.32150
	Schwarzengrund	0.62010	0.32530	2.46900	
		Agona	Enteritidis	Infantis	Schwarzengrund
**Planktonic Ciprofloxacin**	Agona		0.00001	0.00128	0.00079
	Enteritidis	8.64700		0.73640	0.00000
	Infantis	6.02100	1.45000		0.00000
	Schwarzengrund	6.28300	13.63000	11.20000	

Supplementary Table 5. Comparison of persister levels from different culture conditions after exposure to ceftazidime or ciprofloxacin by group of serovars using ANOVA.

Biofilm vs Planktonic						
Serovar	**Ceftazidime**			**Ciprofloxacin**		
	**Sum of squares**	**F-statistic**	**p-value**	**Sum of squares**	**F-statistic**	**p-value**
Agona	0.00001	0.28830	0.60310	0.00002	4.12600	0.06965
Enteritidis	0.00009	4.37500	0.04567	0.00015	17.54000	0.00025
Infantis	0.00009	6.91800	0.02515	0.00001	9.28500	0.01231
Schwarzengrund	0.00001	0.16140	0.70840	0.00001	4.75000	0.09480

Supplementary Table 6. Comparison between SCVs levels from different culture conditions in the same *Salmonella enterica* isolate using ANOVA.

**Biofilm vs Planktonic**			
**Isolate**	**Sum of squares**	**F-statistic**	**p-value**
152	0.02570	0.60890	0.47880
192	0.02154	0.16310	0.70690
393	0.01773	0.44960	0.53920
4SA	0.13481	2.16400	0.21530
S02	0.07014	1.12900	0.34800
S45	0.01048	0.74610	0.43640
S48	0.05711	0.93460	0.38840
S58	0.01174	0.08805	0.78140
S67	0.00017	0.00655	0.93940
S79	0.00490	0.11320	0.75350

Supplementary Table 7. Comparison between ratios of SCVs from different culture conditions after exposure to ciprofloxacin by group of serovars using ANOVA.

Biofilm vs Planktonic			
**Serovar**	**Sum of squares**	**F-statistic**	**p-value**
Agona	466.11600	2.33600	0.15740
Enteritidis	0.02896	0.26920	0.60790
Infantis	0.04496	0.80250	0.39140
Schwarzengrund	935.43500	0.99280	0.37540

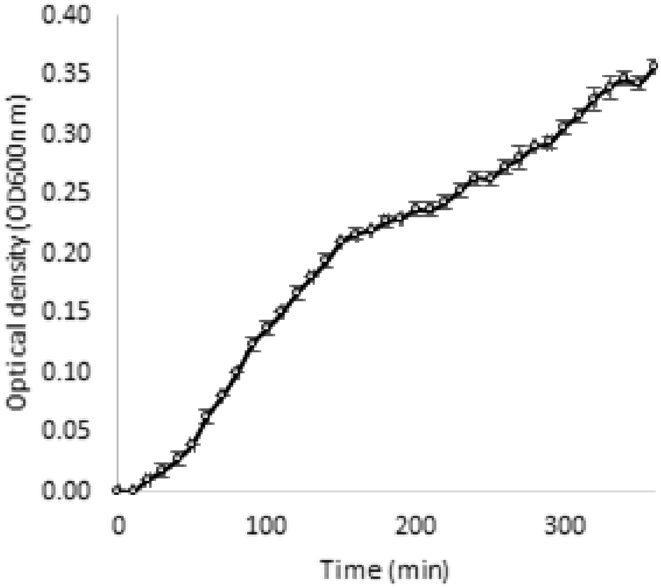

Supplementary Figure S1. Graphical representation of *Salmonella* Infantis growth curve in Luria-Bertani broth (LB) medium. The isolate was diluted 1:30 and the growth at 37 ºC was monitored by measuring the optical density (OD_{600nm}) every 10 min in a SpectraMax® 190 microplate reader. Plotted points represent the mean ± standard deviation of three replicates. The curve fitted show r^2 = 0.92 and a half-life of 6 h.

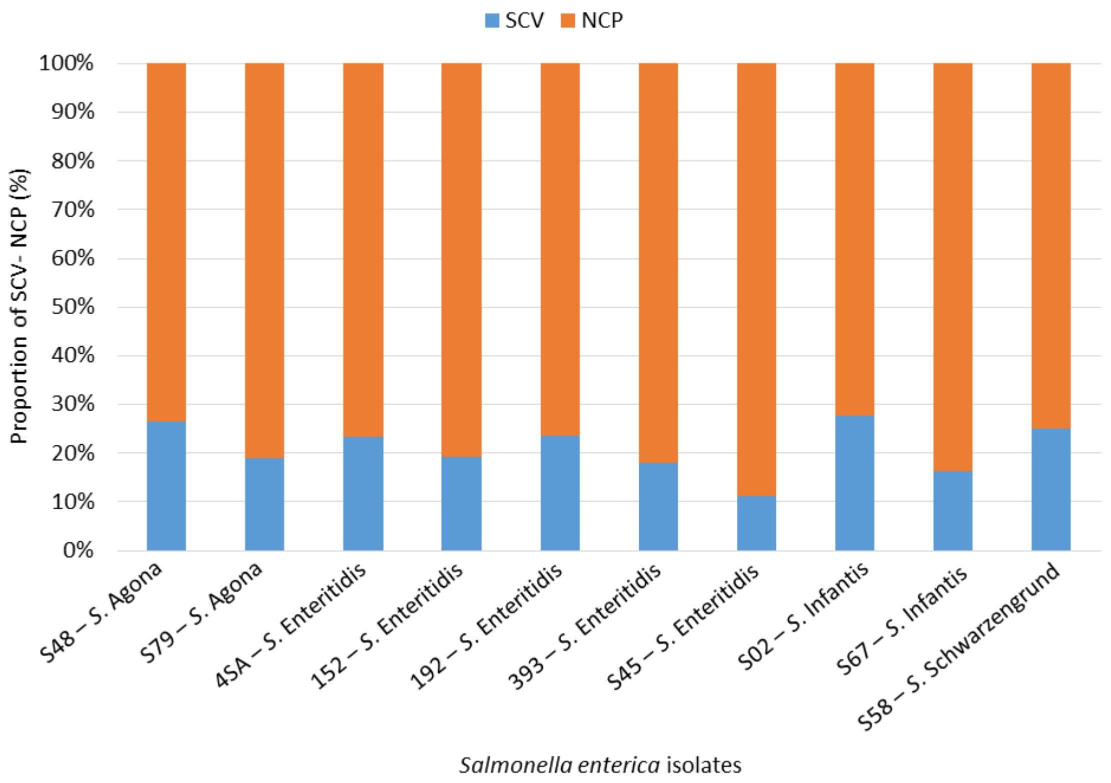

Supplementary Figure S2. Proportions of small colony variants (SCVs) and normal colony phenotypes (NCPs) in each isolate of *Salmonella enterica* exposed to 100-fold MIC of ciprofloxacin. Tukey's test was employed for the statistical analysis and similar ratios were found (p-value > 0.05).

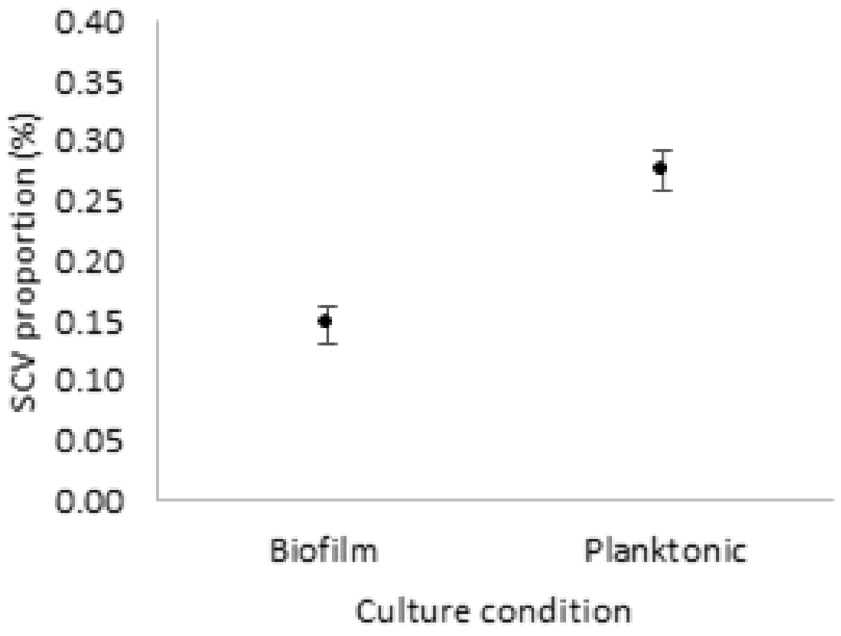

Supplementary Figure S3. Graphical representation of the small colony variants (SCV) proportion found between both culture conditions. The values represent means of three replicates from all isolates and the bars indicate the error standard.

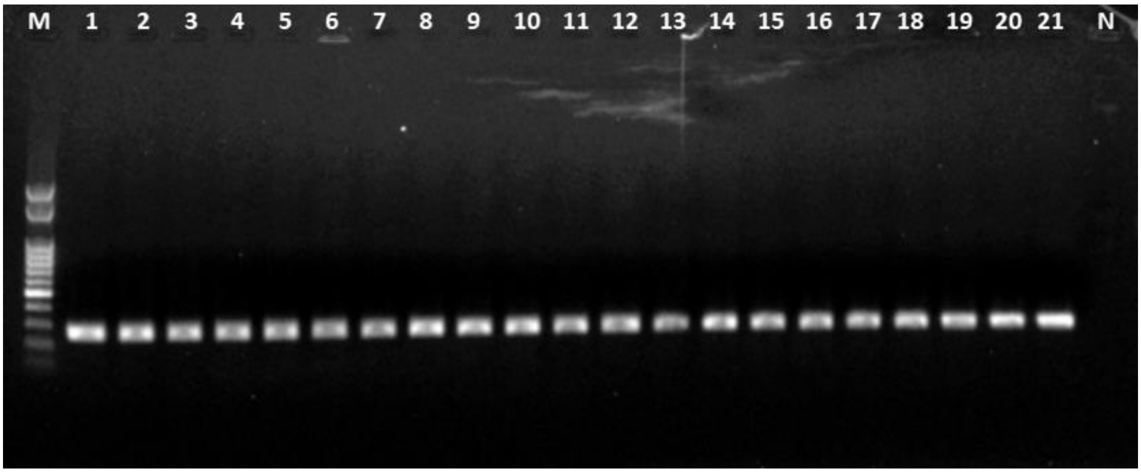

Supplementary Figure S4. Agarose gel electrophoresis of *inv*A gene amplicons from SCV. Lane M: 100 bp DNA Ladder; Lanes 1-21: 284 bp *inv*A amplicons; Lane N: negative control (water was used as sample).

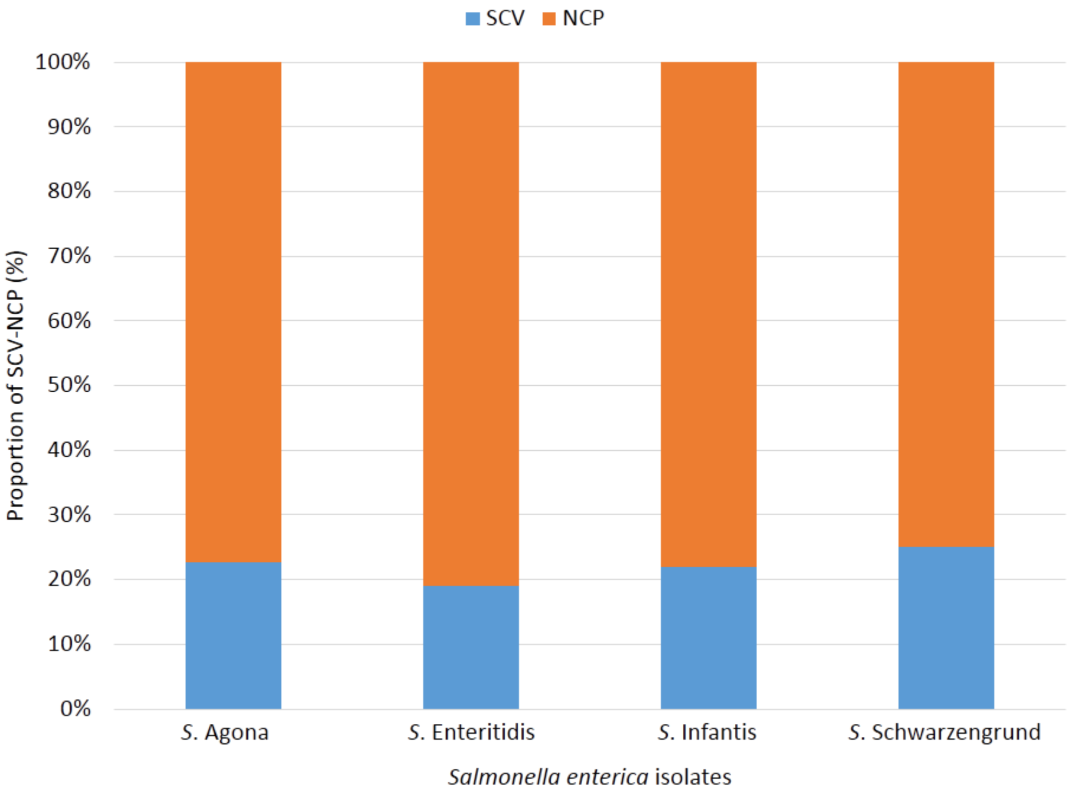

Supplementary Figure S5. Proportions of small colony variants (SCVs) and normal colony phenotypes (NCPs) in each group of *Salmonella enterica* serovar exposed to 100-fold MIC of ciprofloxacin. Tukey's test was employed for the statistical analysis and similar ratios were found (p-value > 0.05).
